# Hyperglycemic Crisis in an Anuric Peritoneal Dialysis Patient with Profound and Symptomatic Hypertonicity

**DOI:** 10.7759/cureus.2566

**Published:** 2018-05-02

**Authors:** James Gibb, Zhi Xu, Mark Rohrscheib, Antonios H Tzamaloukas

**Affiliations:** 1 Department of Medicine, University of New Mexico School of Medicine, Albuquerque, USA; 2 University of New Mexico School of Medicine, Albuquerque, USA

**Keywords:** hyperglycemia, anasarca, hypertonicity, coma, pulmonary edema, peritoneal dialysis, anuria

## Abstract

An anuric peritoneal dialysis patient with diabetes mellitus, congestive heart failure, and anasarca developed severe hyperglycemia with hypertonicity causing profound neurological manifestations after prolonged and continuous use of hypertonic (4.25%) dextrose dialysate. She expired with hypotensive shock from a new myocardial infarction soon after completion of treatment with insulin infusion. The degree of the presenting hypertonicity far exceeded the value expected from the degree of hyperglycemia. We identified prolonged peritoneal dialysis with hypertonic solutions and profound extracellular volume expansion as the causes of the excessive hypertonicity. Hyperglycemia developing in diabetic patients treated for anasarca by peritoneal dialysis after continuous use of hypertonic dextrose dialysate is associated with the risk of excessive hypertonicity with severe clinical manifestations.

## Introduction

Hyperglycemic crises developing in patients with preserved renal function create two categories of diagnostic and treatment challenges. The first category of challenges includes identification and management of the clinical entity – or entities – which caused the hyperglycemia. The second category of challenges consists of body fluid disturbances that can be life-threatening. Major hyperglycemic body fluid disturbances causing severe clinical manifestations and requiring prompt management include extracellular volume deficits, hypertonicity, potassium deficits and maldistribution between the intracellular and extracellular compartments, and acid-base disturbances, mainly metabolic acidosis [1-4]. Other hyperglycemia-induced disturbances, such as magnesium and phosphate deficits, may also need attention and treatment [1]. Osmotic diuresis from glycosuria is a major contributor to body fluid disturbances. Hyperglycemic osmotic diuresis is the only cause of certain disturbances (hypovolemia, potassium deficit), and a major contributor to others (hypertonicity) [2]. Extracellular solute (glucose) gain is the other contributor to hypertonicity [3].

Absence or severe reduction of renal function does not diminish the need for identification and management of the causes of hyperglycemia, but modifies substantially its biochemical and clinical manifestations. Routinely patients on chronic dialysis with severe, or even extreme, hyperglycemia are asymptomatic [5] or exhibit minimal clinical manifestations [6], and have modest hypertonicity [5, 6] and no extracellular volume deficits [6]. Hypertonicity in these hyperglycemic episodes is almost always due exclusively to glucose gain [3]. A rare manifestation of severe hyperglycemia in subjects on dialysis is the development of pulmonary edema, which is corrected after correction of hyperglycemia with insulin [7]. Insulin is the only treatment needed for the great majority of hyperglycemic episodes in patients on maintenance dialysis [8].

We report a patient on continuous peritoneal dialysis presenting with extreme hyperglycemia, pulmonary edema, and severe hypertonicity resulting in dire clinical consequences. This case report illustrates conditions, other than the degree of hyperglycemia, that aggravate the hyperglycemia-induced hypertonicity in oligo-anuric patients.

## Case presentation

An anuric 60-year-old woman with diabetic nephropathy had been treated for four months by continuous ambulatory peritoneal dialysis (CAPD) with four daily exchanges and 2.0 L fill volume. She had a history of coronary artery disease with an anterior myocardial infarction seven months prior to the start of CAPD, followed by severe congestive heart failure with an ejection fraction of 23%. Her dry weight, documented one month after the start of CAPD by absence of both edema and hypertension, was 41.2 kg. Her height was 149 cm. She had not been seen by her CAPD providers for three months because of transportation difficulties when she was admitted with grand mal seizures followed by coma. One week prior to this admission, she communicated by telephone call with one provider. At that time, she reported total body swelling, moderate dyspnea and a body weight exceeding 70 kg. She was advised to use exclusively hypertonic dextrose (4.25%) CAPD dialysate and add two exchanges per day with short (1 hour) dwell times. Information obtained from relatives revealed that in the two days prior to this admission she had omitted her insulin dose because of anorexia and greatly decreased food intake.

On admission, she was comatose with a blood pressure of 180/74 mm Hg, regular pulse rhythm with a rate of 110 beats per minute, and temperature of 36.5 degrees Celsius. Her weight was 63.6 kg. Remarkable findings on physical examination included anasarca detected even in her scalp, a ventricular gallop, and crackles in both lung fields. Chest X-ray confirmed the presence of pulmonary edema and electrocardiogram was unchanged from recent tracings, which were consistent with old transmural myocardial infarction. Laboratory tests on admission revealed extreme hyperglycemia, severe hypertonicity, hypokalemia and respiratory acidosis.

Over the next 30 hours, she received insulin boluses and had frequent (every 2-4 hours) determinations of serum glucose and potassium concentrations. Serum glucose levels decreased progressively, while serum potassium levels remained between 3.1 and 3.4 mmol/L without administration of potassium salts. Blood pressure progressively decreased during treatment. At the end of treatment, she developed frank hypotension (blood pressure 80/60 mm Hg) and increased tachycardia to a pulse rate of 125 mm Hg, while remaining comatose. Chest X-ray revealed worsening pulmonary edema, while electrocardiogram was consistent with a new myocardial infarction and serum levels of cardiac enzymes (creatinine phosphokinase, troponin) were substantially elevated. Subsequently, she developed worsening hypotension and expired within three hours. Table 1 shows laboratory tests at presentation and at the end of insulin treatment.

**Table 1:** Biochemical tests at admission and end of treatment. 1: Serum sodium concentration predicted by Katz’s formula [13] is 164.1 (= 147 + 1.6 x {62.8–3.0}/5.6) mOsm/L. 2: The value of serum tonicity at the end of treatment predicted by Katz’s formula is 331.2 (= 2 x 164.1 + 3) mOsm/L. The observed change in tonicity between presentation and end of treatment is 41.8 (= 356.8–315.0) mOsm/L, while the change in tonicity predicted by Katz’s formula is only 25.6 (= 356.8–331.2) mOsm/L. This comparison confirms a greater than the predicted value ΔTon/ΔGlucose ratio in this patient. Note that the ΔTon/ΔGlucose ratio should be equal during development and correction of hyperglycemia if there are no external losses or gains of fluid and electrolytes during a change in serum glucose concentration. Assuming baseline normal values for serum sodium at 140 mmol/L, glucose at 3.0 mmol/L and tonicity at 283.0 (= 2 x 140 + 3.0) mOsm/L, glucose gain accounted for 41.8 mOm/L of the increase in tonicity at presentation as noted above, while the loss of hypotonic fluids accounted for 32.0 (= 315.0–283.0) mOsm/L. 3: Under room air. 4: With oxygen mask. TCO_2_: Total carbon dioxide content; PAO_2_: Partial pressure of oxygen in arterial blood gases; PACO_2_: Partial pressure of carbon dioxide in arterial blood gases; HCO_3_^-^: Bicarbonate.

Biochemical test	Admission	End of Treatment
Serum glucose, mmol/L	62.8	3.0
Serum glucose, mg/dL	1130	54
Serum sodium, mmol/L	147	156^1^
Serum tonicity, mOsm/L	356.8	315.0^2^
Serum chloride, mmol/L	108	114
Serum potassium, mmol/L	2.9	3.0
Serum TCO_2_, mmol/L	27	27
Arterial blood pH	7.32	7.34
P_A_O_2_, mm Hg	52^3^	114^4^
P_A_CO_2_, mm Hg	50	48
Arterial blood HCO_3_^-^, mmol/L	25.0	25.1

## Discussion

Serum tonicity (effective osmolarity), calculated as 2x(Serum sodium) + Serum glucose in mmol/L, is consistently elevated in hyperglycemia [2, 3]. This case report illustrates two unusual features of tonicity in hyperglycemic episodes occurring in patients on dialysis, excessive hypertonicity with severe neurological manifestations and inconsistent with current guidelines evolution of hypertonicity during correction of hyperglycemia. Tonicity levels exceeding 320 mOsm/L may cause neurological manifestations [5, 6]. Hypertonicity in hyperglycemic episodes developing in patients with preserved renal function has two components, gain in extracellular solute (glucose) and hypotonic body fluid loss through osmotic diuresis [2]. The main cause of severe hypertonicity in hyperglycemic episodes developing in this patient group is the hyperglycemic osmotic diuresis [2].

Hypertonicity in hyperglycemic episodes developing in oligo-anuric patients is routinely modest because its only component is the glucose gain. Loss of hypotonic fluid does not usually occur in these episodes. Furthermore, hyperglycemic hypertonicity is modulated by thirst and water intake and retention in approximately one-third of the patients on dialysis [8]. In a review of published reports of 491 hyperglycemic episodes in patients on chronic dialysis, average values for serum glucose concentration, serum sodium concentration, and serum tonicity were 42.9 mmol/L (772 mg/dL), 127 mmol/L and 298 mOsm/L, respectively [8].

When the only source of hypertonicity is extracellular glucose gain, serum sodium concentration is invariably depressed because of the exit of intracellular water into the extracellular compartment in hyperglycemic episodes [3, 5-9]. Loss of hypotonic body fluids is the only cause of an elevated, or not depressed, serum sodium concentration in the presence of marked hyperglycemia. Severe hypertonicity without hyponatremia in hyperglycemic episodes developing in oligo-anuric patients requires investigation of the site(s) of losses of hypotonic fluids. Such losses can potentially develop in the gastrointestinal tract, the respiratory system, and the skin. Further, hyperglycemic osmotic diuresis may develop in dialysis patients with partially preserved renal function. Finally, loss of hypotonic body fluids can be a consequence of dialysis. Severe hypertonicity was reported in patients on hemodialysis [10] or peritoneal dialysis [11, 12] dialyzed against a dialysate containing high dextrose concentrations. A common characteristic of these observations was that serum sodium concentration was not depressed.

We identified prolonged peritoneal dialysis with hypertonic dialysate as one cause of the severe hypertonicity in the patient presented in this report because of the elevated serum sodium concentration at presentation with extreme hyperglycemia. Loss of water via peritoneal ultrafiltration in relative excess of losses of sodium and potassium was probably aggravated by the two daily exchanges with short dwell times. Early after intraperitoneal instillation of hypertonic dialysate, sodium sieving results in lower sodium concentration in the dialysate than in plasma. The lowest dialysate sodium concentration is observed approximately one hour after intraperitoneal infusion of the dialysate [13].

A second component of the excessive hypertonicity of our patient at presentation with hyperglycemia was the presence of anasarca. The influence of anasarca on the degree of hypertonicity was identified by analyzing the rates of increase in serum sodium and decline in tonicity during treatment. Throughout treatment, the patient received no fluids and had no detectable fluid losses. Under these conditions, serum sodium concentration increases through osmotic transfer of extracellular fluid into the intracellular compartment as serum glucose concentration decreases [8, 9]. We compared the rate of change in serum sodium concentration over the rate of change in serum glucose concentration observed in the patient presented in this report and the corresponding rates predicted by Katz [14] and computed in an analysis of 43 episodes of severe hyperglycemia treated with infusion of insulin [15]. Katz calculated an increase in serum sodium concentration of 1.6 mmol/L for each 5.6 mmol/L (100 mg/dL) decrease in serum glucose concentration [14]. This calculation assumed that the changes in sodium concentration and tonicity result only from a decrease in extracellular glucose content. Anuric hyperglycemia treated only with insulin allows testing of Katz’s theoretical calculations. In the study analyzing tonicity changes during treatment of hyperglycemia in patients on dialysis with insulin, mean increase in serum sodium concentration was 1.5 mmol/L per 5.6 mmol/L decrease in serum glucose concentration [15]. This value is almost identical to the value predicted by Katz. The rate of rise in serum sodium in our patient was only 0.84 (= 5.6x {156–147]/{62.8–3.0}) mmol/L per 5.6 mmol/L.

The change in tonicity (ΔTon) during development or correction of hyperglycemia is the difference between the changes in the osmotic contributions of the changes in serum glucose (ΔGlucose) and sodium concentrations (ΔNa), or ΔTon = ΔGlucose – 2xΔNa [3]. During a change in serum glucose concentration, the lower the ΔNa is for the same ΔGlucose, the higher the ΔTon will be. For example, ΔTon values for each 5.6 mmol/L ΔGlucose are 2.2 (= 5.6–2 x 1.6) mOsm/L from Katz’s prediction [14] and 3.93 (= 5.6–2 x 0.84) mOsm/L in the patient of this report. The low value of the ratio ΔNa/ΔGlucose observed during treatment in our patient accounts for part of the severity of hypertonicity at presentation, is not the result of fluid losses or gains, and requires an explanation.

The reason for the low rate of change in serum sodium concentration and high rate of change in tonicity during treatment of the hyperglycemic episode in our patient was the profound expansion of extracellular volume. The main determinants of the changes in tonicity in hyperglycemic episodes occurring in oligo-anuric patients without losses of hypotonic fluids are the degree of hyperglycemia and the state of extracellular volume [16]. For the same degree of hyperglycemia, higher levels of extracellular volume result in lower rates of decrease in serum sodium concentration and higher rates of rise in tonicity. These theoretical predictions are based on the fact that after a change in body glucose content the change in serum glucose concentration is determined by extracellular volume while the change in tonicity is determined by total body water [3]. This relationship is expressed as ΔTon/ΔGlucose = (Extracellular volume)/(Total body water) [3]. The value ΔTon/ΔGlucose was 0.71 (= 3.93/5.6) mOsml/L per mmol/L in our patient. We estimated the fraction (Extracellular volume)/(Total body water) in this patient as shown below.

Total body water at our patient's dry weight calculated by the Watson formula [17] was 23.99 (= -2.097 + 0.1069 x 149 + 0.7466 x 41.2) L. Assuming that her euglycemic extracellular volume at dry weight was 40% of total body water [18], this volume was 9.6 (= 0.4 x 23.99) L. The weight excess of 21.8 (= 63.6–41.8) kg at presentation represented a gain in extracellular volume. Therefore, euglycemic extracellular volume at the admission weight would be 31.4 (= 9.6 + 21.8) L, while total body water was 45.79 (= 23.99 + 21.8) L. From these values, an (Extracellular volume)/(Total body water) ratio of 0.69 (= 31.4/45.79) is calculated, which is almost identical to the observed ΔTon/ΔGlucose value of 0.71. The calculations of total body water and extracellular volume are subject to substantial errors [18]. Nevertheless, the comparison of the ratios ΔTon/ΔGlucose and (Extracellular volume)/(Total body water) provides some support to our conclusion that extracellular volume expansion was the cause of the difference in tonicity changes during correction of hyperglycemia between the value observed in the patient of this report and the predicted [14], and usually observed [15], values.

Two last points of interest in this case report are the evolution of serum potassium concentration during treatment and the presenting acid-base disturbance. A decrease in serum potassium concentration during treatment is routinely observed during treatment of anuric hyperglycemia as a result of direct action of insulin on cell membranes and correction of hypertonicity [19]. Infusion of potassium salts was held in our patient, who presented with hypokalemia, because of successive serum potassium values exceeding the value at presentation. It is not clear whether this lack of decrease in serum potassium concentration was due to extracellular volume contraction during treatment, concurrent state of a catabolic state causing an increasing release of potassium from cells, or other process. Regardless of the developments in this patient, serum potassium should be monitored during treatment of hyperglycemia with insulin in oligo-anuric patients. Patients in this group have often hyperkalemia at presentation and need potassium replacement rarely if ever [19]. Potassium replacement in anuric patients with hypertonicity requires special attention because it carries the risks of hyperkalemia and further increases in tonicity. Finally, respiratory acidosis, which was noticed in our patient at presentation and throughout her hospital course, was reported in 11 of 46 patients with pulmonary edema studied by Aberman and Fulop [20]. Of note is that respiratory acidosis was not a sign of severity of the pulmonary edema in this study [20].

## Conclusions

Symptomatic hypertonicity from severe hyperglycemia without hyponatremia in oligo-anuric patients calls for search for conditions causing loss of hypotonic body fluids. Presence of pronounced edema is another risk factor for excessive hypertonicity in oligo-anuric patients developing severe hyperglycemia. Peritoneal dialysis in diabetic patients with the exclusive use of hypertonic solutions is associated with significant risks for hyperglycemia and hypertonicity, especially in the presence of pronounced edema, and should be performed in a hospital setting with frequent monitoring of the clinical status of the patient and of serum glucose, sodium, and potassium concentrations.
